# The application of artificial technology in pediatric pyeloplasty the efficacy analysis of robotic-assisted laparoscopic pyeloplasty in the treatment of ureteropelvic junction obstruction

**DOI:** 10.3389/fped.2023.1209359

**Published:** 2023-09-15

**Authors:** Zhongli Hu, Shan Chen, Zhihong Wang, Di Xu, Xiaolang Zhang, Yang Lin, Lin Zhang, Jianbin Wang, Lizhi Li

**Affiliations:** ^1^ Affiliated Hospital of Putian University, Putian, China; ^2^ Department of Laboratory, Fuzhou Second Hospital, Fuzhou, China; ^3^ Department of Hematology, Provincial Clinical Medical College, Fujian Medical University, Fuzhou, China; ^4^ Department of Pediatric Surgery, Provincial Clinical Medical College, Fujian Medical University, Fuzhou, China

**Keywords:** UPJO, RALP, LP, Pediatric, ERAS

## Abstract

**Objective:**

To investigate the clinical effect of the da Vinci robotic-assisted laparoscopic pyeloureteroplasty (RALP) in treating pediatric ureteropelvic junction obstruction (UPJO).

**Methods:**

We retrospectively analyzed clinical data from 32 children with UPJO who suffered from RALP in our hospital from October 2020 to February 2023, compared with those treated with common laparoscopy at the same time. After the establishment of the robotic arm, a mesenteric approach was performed after entering the abdominal cavity to focus on the lesion site. The dilated renal pelvis was then cut and the stenotic ureter was removed; the anastomosis and the incision were sutured by layer.

**Results:**

A total of 62 children (44 boys and 20 girls) with a median age of 14 months (ranging from 3 to 38 months) were included. All 62 cases had hydronephrosis caused by unilateral UPJO, and the surgery was successfully completed without conversion to open. All intraoperative blood losses amounted to less than 10 ml. In the RALP group, the average operative duration was 131.28 min (ranging from 108 to 180 min). The average catheter time was 3.66 days (ranging from 2 to 7 days). The average hematuria time was 3.84 days (ranging from 2 to 6 days). The average postoperative hospital stay was 7.8 days (ranging from 6 to 12 days). The average hospitalization costs were 59,048.31 yuan (ranging from 50,484 to 69,977 yuan). The double-J tube was removed 1 month after surgery. Only one patient suffered from complications, developing a urinary tract infection 4 weeks after surgery, and was cured with the administration of oral cefaclor anti-inflammatory drugs for 3 days. All patients were followed up for 2–28 months, with a median follow-up time of 12 months. The thickness of the renal cortex was increased after surgery [(1.95 ± 0.24) vs. (4.82 ± 0.50)] cm, and the isotope renograms revealed a definite recovery of the split renal function [(28.32 ± 1.95) vs. (37.01 ± 2.71)]%.

**Conclusion:**

The robotic-assisted laparoscopic pyeloureteroplasty (RALP) in the treatment of children with upper ureteral obstruction has overall clinical efficiency. With technological advancements and an increased number of experienced surgeons, robotic surgery may become a new trend in surgery.

## Introduction

Ureteropelvic junction obstruction (UPJO) is the major etiology of congenital hydronephrosis in children with upper urinary tract obstruction (1). Typically, the junction between the renal pelvis and ureteral presents a funnel shape, and the pressure stimulation of urine from the renal pelvis facilitates the anterograde peristalsis of the ureter. UPJO is defined as an intrinsic stenosis at the ureteropelvic junction, while the urine of the renal pelvis hardly enters the upper ureter (2), which is often correlative with the anatomic defects of the ureteropelvic junction, such as the slender ureteropelvic junction, the high ureteropelvic junction, the valve or polyps of the ureteropelvic junction, and vascular compression (3), which induce a progressive dilatation of the renal collecting system and subsequent impairment of renal function (4). By means of ultrasonography, 85%–90% of severe hydronephrosis in infants is caused by UPJO (5), evaluated by increased anteroposterior diameter (APD) of renal without ureter dilatation.

The degree of anatomic defects and symptoms should be considered in the clinical intervention of children with unilateral UPJO (6). Although most younger infants without adverse effects can be managed conservatively, severe hydronephrosis due to UPJO requires early surgical intervention. Dismembered pyeloplasty is the standard surgical approach for UPJO, and the reconstruction of the ureteropelvic junction can be achieved through minimally invasive laparoscopic pyeloplasty (LP) (7). Robotic-assisted laparoscopic pyeloplasty (RALP) has gradually gained acceptance since the initial introduction of LP in 1993 (8).

In this study, we retrospectively analyzed 32 cases of UPJO treated with RALP in our hospital from October 2020 to February 2023 to investigate the clinical efficiency of RALP in treating pediatric UPJO.

## Materials and methods

### Clinical information

We retrospectively analyzed clinical data from 62 children diagnosed with UPJO in our hospital from October 2020 to February 2023. In total, 32 patients underwent robotic-assisted laparoscopic pyeloplasty (RALP) and the other 30 patients underwent traditional laparoscopic pyeloplasty (LP). The details of the patients are presented in Table 1. Before the surgery, we informed the patients’ families about the benefits and drawbacks associated with both surgical options mentioned above. The final decision was made by the families based on their preferences. All the operations were conducted by the same experienced surgical group. The preoperative evaluation of all patients demonstrated no adverse surgical contraindications.

**Table 1:** Comparison of general information and preoperative conditions of the two surgical groups.

Groups		RALP	LP	*t/X^2^*	*P* value
Sample number		32	30	–	
Gender	Male (%)	22 (68.75)	20 (66.67)	–	
	Female (%)	10 (31.25)	10 (33.33)		
Age (month, $\bar{x}\pm s$)		16.91 ± 10.08	16.80 ± 9.23	0.043	0.966
Lesion location	Right (%)	15 (46.88)	14 (46.67)	–	
	Left (%)	17 (53.12)	16 (53.33)		

RALP, robotic-assistant laparoscopic pyeloplasty; LP, laparoscopic pyeloplasty.

*t/X*^2^, a *χ*^2^ test with continuity correction.

The inclusion criteria were as follows: (1) fetal hydronephrosis, with postnatal follow-up urinary ultrasound revealing a progressive increase in nephroureteral dilatation; (2) symptoms of hydronephrosis (abdominal mass, abdominal pain, repeated urinary tract infections, etc.); (3) diuretic renal radionuclide imaging showing a persistent decrease in split renal function <40% (2); (4) hydronephrosis after pyelostomy; (5) intact clinical and follow-up data.

The exclusion criteria are as follows: (1) patients with acute urinary tract infections; (2) patients with dysfunctions of the heart, liver, lung, or other organs; (3) patients with abdominal surgery history leading to extensive abdominal adhesions; (4) patients with primary vesicoureteral reflux and ureterovesical junction obstruction (UVJO); (5) secondary operation, bilateral operation or conversion to open surgery; (6) patients with duplex kidney, solitary kidney, or split renal function <10%; (7) defective clinical and follow-up data.

### The instruments of the robotic and laparoscopic surgical device

The instruments of the robotic and laparoscopic surgical device include (1) the da Vinci Endoscopic Instrument Control System (IS4000, da Vinci Si), which contains the endoscopic surgical instrument control system (main console), robotic arm system (motion arm and camera arm), and three-dimensional imaging video system and (2) the Storz pediatric laparoscopic instruments (SYZBA-2010142, 7220BA/26005BAK).

### The placement of trocars and robotic arms

**Figure 1:**
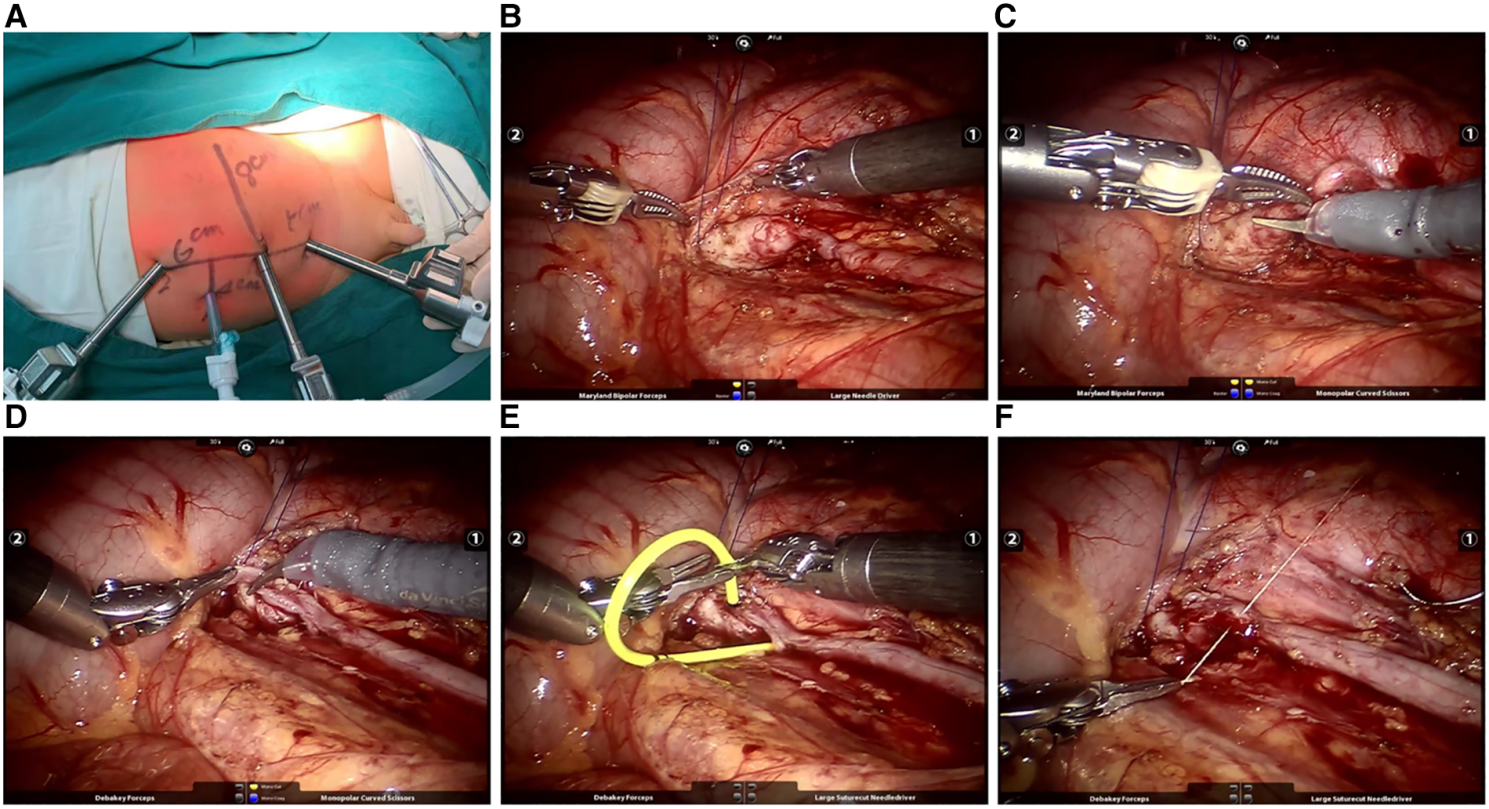
(**A**) Location of robotic trocars; (**B**) tow of ureterpelvis; (**C**) dissection of UPJO; (**D**) clipping of dilated ureter; (**E**) placement of double-J catheter; (**F**) reconstruction of funnel-shaping UPJ.

A triporate-plus-one approach was performed, and a robotic surgical system device was docked: an 8.5 mm trocar was placed around the umbilicus to connect the camera arm; a 5 mm trocar was placed at the junction of the transverse striation of the hypogastrium and the ventral midline to connect manipulator 2 with the dissecting forceps or grasping forceps for clamping and assisted traction. An 8 mm trocar was placed at the symmetry point of manipulator 2 trocar at the upper abdomen to connect manipulator 1 with the monopolar electric hook and needle holders for dissociating, cutting, and suturing. The distance between the functional holes and the umbilicus was 3–5 cm. Additionally, a 3 mm assisted trocar was placed at the opposite anterior axillary line parallel to the lesion kidney (Figure 1A).

### Da vinci robotic surgery procedure

After endotracheal intubation anesthesia, the patient was positioned in a healthy lateral decubitus position, tilted at an angle of 45°–80°. The peritoneal approach was performed as above after the establishment of pneumoperitoneum (8–14 mmHg). On the left side, the dilated renal pelvis and ureter at the ureteropelvic junction were exposed through the mesenteric approach, while on the right side, the colon was mobilized at the hepatic flexure. Special attention was paid to the relationship between the kidney, colon, and mesenteric vessels. The intestines were repositioned to expose the descending mesocolon. The mesentery and posterior peritoneum were unfolded medial to the genital vein, and the perirenal fascia was dissected to expose the dilated renal pelvis and upper ureter. After gently retracting the renal pelvis and ureter with a 3-0 absorbable suture (Figure 1B), the curved and stenotic ureter was excised from the dilated renal pelvis (Figure 1C). A 1.5 cm longitudinal incision was made in the distal ureteral wall of the stenotic segment, and the lower angle of the renal pelvis flap was sutured to anchor at the distal incision with a 6-0 absorbable suture. The posterior wall of the anastomosis was then continuously sutured (Figure 1D). A double-J tube was placed anterogradely through a 5 mm trocar to reach the anastomosis (Figure 1E). Cystoscopy was used to ensure the pigtail in the bladder was visible and not obstructing the ureteric orifice. Subsequently, the ureteropelvic junction was cut to further trim the renal pelvis, and the anterior wall of the anastomosis and the excess renal pelvis opening were continuously sutured (Figure 1F). The wound was rinsed with normal saline, and the mesenteric hiatus was intermittently sutured using a 6-0 absorbable suture. Finally, the abdomen was closed in layers after the placement of abdominal drainage.

### Laparoscopic pyeloplasty procedure

In the transperitoneal approach, three longitudinal incisions of 1 cm were respectively made at the umbilicus, 3 cm away from the subxiphoid and the McBurney's point (right lesion) or anti-McBurney's point (left lesion), for the docking of the laparoscopic device. A Veress needle was placed at the umbilical incision to establish pneumoperitoneum and place the laparoscope, and the operating instruments (hooks, scissors, needle holders, etc.) were placed at the other two incisions. The surgical procedure was the same in the robotic group.

### The observation of peri-operation records

(1) The gender, age, lesion kidney, hydronephrosis degree classified by the society for Fetal Urology (SFU) (9), preoperative thickness of lesion renal cortex and split renal function between the two groups were recorded. (2) The operative duration, intraoperative bleeding volume, postoperative hospital stay, incision healing time, catheter time, fasting time, and hospital costs between the two groups. (3) The postoperative complications (anastomotic leakage, urinary tract infection, etc.). (4) Six months after removing the ureteral stent, urinary ultrasound and diuretic renal radionuclide imaging were performed to record the postoperative thickness of the renal cortex and the split renal function. The increase in the thickness of the renal cortex and the split renal function were calculated and compared to preoperation. The total length of the skin surface scar was measured (with the same steel ruler); the Vancouver Scar Scale was used to score the operative scar (the higher the score, the more obvious the scar); and a Likert Scale was used to identify the satisfaction of the child's families regarding the incision recovery, with scores of 5-1 corresponding to very satisfied, satisfied, general, dissatisfied, and very dissatisfied, respectively.

### Postoperative follow-up

After surgery, cefuroxime was intravenously infused for 3–6 days, and cefaclor was orally administered later for anti-infection. The patient could be discharged with stable vital signs, normal diet, and no incision infection or urine leakage. The double-J stent was removed under a cystoscope 1 month after discharge. Urinary ultrasound was reviewed 3 months post-operation, and isotope renogram was reviewed 6 months post-operation. Normally, urinary ultrasound was reviewed every 6 months or 1 year and isotope renogram was reviewed once a year thereafter to observe the dilatation of the pelvis and the thickness of the renal cortex. The valid surgery was judged by the alleviation of hydronephrosis, and the split renal function that was improved or stabilized at the preoperative level.

### Statistical analysis

Statistical analysis was performed using SPSS 22.0 software. When measurement data conformed to a normal distribution, data were expressed as mean ± SD, and an independent sample *t*-test was used to compare data from the two groups. Enumeration data was expressed as a rate (%), the comparison between the two groups used the *X*^2^ test, and the exact *X*^2^ used the Fisher method. *P *< 0.05 was considered statistically significant.

### Ethical statement

The study was approved by the Fujian Province Hospital ethics committee (ethics approval number: K2020-10-362). In addition, informed consent was obtained from the legal guardians of all patients prior to the associated procedure in the study.

## Results

A total of 62 children (44 boys and 20 girls) with a median age of 14 months (ranging from 3 to 38 months) were included. All 62 cases had hydronephrosis caused by unilateral UPJO, and their surgeries were successfully completed without conversion to open. All intraoperative blood losses amounted to less than 10 ml. In the RALP group, the average operative duration was 131.28 min (ranging from 108 to 180 min). The average catheter time was 3.66 days (ranging from 2 to 7 days). The average hematuria time was 3.84 days (ranging from 2 to 6 days). The average postoperative hospital stay was 7.8 days (ranging from 6 to 12 days). The average hospitalization costs were 59,048.31 yuan (ranging from 50,484 to 69,977 yuan) (Table 2). The double-J catheter was removed cystoscopically 1 month after surgery. All the patients were followed up for 2–28 months, with a median follow-up period of 12 months. No patients had postoperative complications, except for one, who developed a urinary tract infection 4 weeks after surgery, which might have been caused by the indwelling of the double-J stent; they received symptomatic treatment consisting of oral cefaclor anti-inflammatory drugs for 3 days. The thickness of the renal cortex increased in all 32 patients after surgery [(1.95 ± 0.24) vs. (4.82 ± 0.50)] cm, and isotope renograms revealed the prospective recovery of split renal function [(28.32 ± 1.95) vs. (37.01 ± 2.71)]% (Table 3). Compared with LP, the data of the RALP group showed a shorter duration of operation, postoperative catheter, and hematuria and recovery but similar clinical efficiency.

**Table 2:** Comparison of intraoperative and postoperative conditions of the two surgical groups.

Groups	RALP	LP	*t*/*X*^2^	*P* value
Sample number	32	30	–	
Operation time (min, $\bar{x}\pm s$)	131.28 ± 20.48	176.03 ± 27.95	−7.222	0.000
Catheter time (d, $\bar{x}\pm s$)	3.66 ± 1.26	6.10 ± 1.63	−6.638	0.000
Hematuria time (d, $\bar{x}\pm s$)	3.84 ± 1.08	6.43 ± 1.99	−6.411	0.000
Postoperative discharge time (d, $\bar{x}\pm s$)	7.78 ± l.84	9.47 ± 2.15	−3.323	0.002
Cost (yuan, $\bar{x}\pm s$)	59048.31 ± 5102.08	21716.27 ± 3642.84	32.961	0.000

RALP, robotic-assistant laparoscopic pyeloplasty; LP, laparoscopic pyeloplasty.

*t/X*^2^, a *χ*^2^ test with continuity correction.

**Table 3:** Comparison of preoperative and postoperative conditions of RALP.

Record		preoperation	6 months after operation	*t*/*X*^2^	*P* value
Renal cortical thickness (cm, $\bar{x}\pm s$)	RALP	1.95 ± 0.24	4.82 ± 0.50	−29.045	0.000
	LP	1.93 ± 0.23	4.73 ± 0.49	−28.256	0.000
Split renal function (%, $\bar{x}\pm s$)	RALP	28.32 ± 1.95	37.01 ± 2.71	−14.721	0.000
	LP	27.92 ± 1.79	36.29 ± 2.67	−14.276	0.000

RALP, robotic-assistant laparoscopic pyeloplasty; LP, laparoscopic pyeloplasty.

*t/X*^2^, a *χ*^2^ test with continuity correction.

## Discussion

The prophase intervention of neonatal hydronephrosis required the accurate identification of anastomosis defects, as they can cause progressive kidney damage. The identification of ureterovascular hydronephrosis can prevent patients from unnecessary pyeloplasty (10). Primary hydronephrosis usually presented unilaterally and developed into ureteropelvic or ureterovesicle junction defects (11). Few cases of unilateral hydronephrosis associated with UPJO were accompanied by deficiency of split renal function or progressive hydronephrosis (12), indicative of pyeloplasty. Patients with primary adverse dilatation of the renal pelvis and split renal function could be re-considered after null conservative intervention.

Since Anderson and Hynes first reported the open dismembered pyeloplasty (Anderson-Hynes’ pyeloplasty) (13), it had become the gold standard for the treatment of UPJO. By 1995, Peters et al. completed the first LP in children (14), and Anderson-Hynes’ pyeloplasty has since entered the endoscopic era. However, there were adverse matters accompanying the development of LP. On one hand, lacking of stereoscopic sensation in the two-dimensional planar imaging and tough manipulation of laparoscopic device, result in disability of handling subtle procedure. Surgeons with poor laparoscopic experience might struggle to achieve steady quality in dissection and suture, especially in children with a small renal pelvis or renal meniscus rotation, leading to a higher risk of postoperative complications. On the other hand, due to the limitations of the pediatric abdominal cavity, it was difficult to achieve adequate exposure of the surgical field. Additionally, children were featured with poor tolerance to surgery and pneumoperitoneum, which invisibly increased the difficulty of LP. The minimally invasive surgery was defined again with the development and application of da Vinci robotic technology.

The application of da Vinci robotic technology in adult surgery has been deemed safe and feasible. However, da Vinci robotic surgery in children has been slow to progress due to the conflict between the adequate manipulated space of robotic devices and the limited cavity of the pediatric body (15). Nevertheless, pediatric surgery for urinary defects could be assisted to completion by the robotic system (16). The robotic system provides three-dimensional, magnified, accurate, and 10-fold high-definition imaging to assist surgeons in comfortably removing stenotic ureteral lesions. The robotic manipulator can be stretched out in a limited cavity with little vibration but accurate calibration (17). This superiority over laparotomy or LP makes robotic-assisted pyeloplasty an appealing option. The unique 3D high-definition magnified imaging of the robotic system offers more vivid exposure of the renal pelvis and ureter, enabling surgeons to perform subtle separation and minimize dissection damage (18).

The dissection of the dilated renal pelvis and uteropelvic anastomosis were crucial steps in these surgeries and can be associated with postoperative complications. The fine filtration ability of the robotic manipulator allows surgeons to focus on the precision of these procedures, achieving meticulous handling. In the RALP group, no patients developed postoperative complications, except for one, who suffered from urinary tract infection (UTI) 4 weeks after surgery. Due to the capabilities of the robotic manipulator, the Enhanced Recovery After Surgery (ERAS) protocol was implemented more in the perioperative nursing of the pediatric patients. The postoperative hospital stay of the children in our study who underwent RALP was 6–12 days, with an average one of 7.8 days, which was feasible compared with the general discharge of time approximately 9 days (19).

The robotic system provided more appropriate conditions for surgeons in prolonged operations. With the same surgical procedure as LP, the duration of robotic surgery could be shortened further as surgeons gain experience and with the tacit cooperation of assistants and nurses and the proficient docking of the robotic device (20). In our study, the initial surgical duration of RALP was between 240 min and 255 min, respectively, and the duration was controlled in 2 h by surgeons with proficiency. RALP in children reduced postoperative pain and the duration of their hospital stay (21), which was consistent with the advanced accuracy, finer anastomosis, more comprehensive visual field, and more minimally invasive nature of this surgical approach. The minimally invasive surgery resulted in a lower local inflammatory response, less exudate, and faster recovery of gastrointestinal function, which may be responsible for the decreased exudation, fasting time, and discharge time of robotic surgery. The difference in CV time duration and hematuria time between the two groups might be associated with the three-dimensional, magnified visualization and enhanced dexterity, precision, and range of motion of the surgical system.

With the development of technology, RALP has been steadily optimized for shorter surgery overall and fewer postoperative complications. The retroperitoneal approach is more applicable to exposing the ureteropelvic junction and clipping the UPJO (22). The traditional dismembered pyeloplasty was repealed to adapt to the ureteropelvic junction reconstruction of long-segment stricture (23). The tonic anastomosis and anterograde peristalsis could be satisfied by the tubular of the pelvic double-flap, which broadened the cavity of UPJ (24). The shaping of the funnel pelvis ensured the motile peristalsis and feasible diameter of the reforming ureter. Thus, the favorable anastomosis and minimal invasion of RALP were more adequate for the severe hydronephrosis of pediatric UPJO.

The anatomical differences and difficulties between RALP and LP are as follows. (1) Reconstruction of the renal pelvis and ureter is a crucial step in pyeloplasty, especially the cutting length of ureter and suture of lowest renal pelvis with the cutting ureter. The robotic system in RALP provides improved visualization and instrument articulation, which aids in the dissection and reconstruction of these structures. While LP uses long, rigid instruments with limited degrees of freedom, which can make fine dissection and suturing maneuvers more challenging. (2) RALP allows for high-definition 3D visualization in the identification and mobilization of the upper ureter and renal pelvis. LP relies on 2D visualization using a laparoscope, which can limit depth perception and make it more challenging to identify fine anatomical structures accurately. (3) Both RALP and LP encounter limited space within the abdomen and pelvis, making it challenging to access and manipulate the surgical site. However, RALP benefits from a robotic system that offers enhanced dexterity and range of motion, allowing for more precise movements in a constrained space.

The limitations of RALP are as follows. (1) The operation cost is higher than that of laparoscopic surgery, which limits its popularization in clinical treatment. (2) At present, the da Vinci robotic system is difficult to replace the patient's position intra-operation. The da Vinci Xi might be capable of overcoming this problem (25). (3) The application of RALP in infants less than 1 month of age is limited. (4) When 6-0 PDSII and other thinner sutures are used for continuous suture, the lack of tactile feedback results in difficulty in suture and knotting (26).

## Conclusion

While both RALP and LP have their respective anatomical difficulties, RALP generally offers advantages in terms of improved visualization, enhanced dexterity, and better instrument maneuverability. These benefits can be particularly valuable when performing delicate procedures such as pyeloplasty, allowing surgeons to overcome anatomical challenges and achieve greater precision in dissection and reconstruction. It's important to note that the specific challenges can vary depending on factors such as patient anatomy, surgeon experience, and individual surgical techniques. Surgeons may adapt their approaches to address these difficulties and achieve successful outcomes using either RALP or LP for pyeloplasty.

## Data Availability

The original contributions presented in the study are included in the article/Supplementary Materials, further inquiries can be directed to the corresponding author.
